# HPV negative cervical cancers and primary HPV screening

**Published:** 2018-06

**Authors:** WAA Tjalma

**Affiliations:** Multidisciplinary Breast Clinic, Gynecological Oncology Unit, Department of Obstetrics and Gynecology, Antwerp University Hospital, University of Antwerp, Wilrijkstraat 10, 2650 Edegem, Belgium.

**Keywords:** HPV, negative, vaccination, screening, primary, prevention, cytology, dual staining, diagnostic cytology, morbidity, mortality, cervical cancer

## Abstract

More than 25 years ago it was established that a HPV (Human Papilloma Virus) infection was the causal factor for cervical cancer. Based on this discovery HPV vaccines were developed and primary HPV screening proposed. The impact of 10 years prophylactic HPV vaccination with the bivalent and quadrivalent vaccines has been tremendous. There is a reduction of HPV infections 16/18, 31, 33 and 45 of respectively 89%, 94%, 79% and 83%. High grade lesions have been reduced by 85% and warts by 90%. Within 20 to 30 years a reduction in cervical cancer incidence, by 70-80%, is to be expected. The 9 valent HPV vaccine, which was introduced last year and is reimbursed for girls between 12 – 19 years, is expected to increase the figures by 14 to 18%.

Recently, doubt has been created regarding primary HPV screening. Since 2017, the annual screening report in Belgium suggests that 15% of the cervical cancers were HPV negative. Previous published data in Belgium (period 2001 - 2008) showed that the number of HPV negative tumors is less than half of the suggested figure (7%). Frequent reasons for false negative HPV tumors are the used HPV testing methods and the misclassification of endometrial cancers or metastasis as cervical cancers. Other explanations are the loss of HPV expression and the existence of cervical cancers independent of HPV. The incidence of HPV negative tumors doesn’t give any information about the performance of primary HPV screening. Data from randomized controlled trials are very clear: if a woman has a normal cytology and no HPV infection or normal cytology and a HPV infection, then her chance of developing a CIN3 + lesion after 5 years is, respectively, 0,2% and 6%. In Belgium, primary HPV screening with dual-stain cytology triage would considerably reduce the incidence (36%) and mortality (40%) of cervical cancer. There is necessity to improve the screening as we are entering an era of vaccinated women who will get screened. Standardized high quality HPV testing is the key stone for improvement. HPV screening preferable with triage markers is superior to cytology, despite the fact that there are HPV negative cancers. The fact that there are HPV negative cancers should not undermine all idea’s regarding primary HPV screening.

## Introduction

Cervical cancer remains a major health issue in Belgium with 4070 in situ cancers and 468 invasive cervical cancers in the 2013-2014 period (Annual Report, 2017). Primary and secondary prevention, however, has the potential to make cervical cancer a historical disease (Tjalma et al., 2004; 2005).

The primary prevention is the prophylactic HPV vaccination. At present, there are three prophylactic HPV vaccines: Gardasil, Cervarix and Gardasil 9. The school-based vaccination program in the Flemish part of Belgium has a coverage of 91% and in the French Community the coverage is around 36% (Tjalma et al, 2019). Recent long-term data in countries with a high vaccination coverage (> 80%) showed a reduction in targeted HPV infections with 90% and high-grade lesions with 85% (Garland et al., 2016; Kavanagh et al., 2017). Within 20 to 30 years it is expected that the incidence of cervical cancer will decrease by 70 to 80% (Tjalma, 2015). The 9 valent HPV vaccine, which was introduced last year and is reimbursed for girls between 12 – 19 years old, is expected to increase the figures by 14 – 18%.

We still have to continue cervical cancer screening because not all women are vaccinated and the vaccine does not protect against all types of cervical cancer. Cervical cancer screening should shift from primary cytology to primary HPV screening (Tjalma, 2014). Ideally, diagnostic cytology should be performed. This would reduce the incidence of cervical cancer by 36% and the annual mortality by 40% (Tjalma, 2017; Tjalma et al., 2017). Despite the fact that we believe that almost all cervical cancers are due to a HPV infection, we do not always find HPV in women with cervical cancer. The recently published annual cervical cancer screenings report in Belgium suggested that 15% of cervical cancers were HPV negative (Annual Report, 2017). This high figure created confusion on whether or not primary HPV screening is the best way forward. It is important to realize that primary HPV screening will, like every cancer screenings test, not detect all precancers and cancers. The present article will address the issue of HPV negative cancers and give a possible explanation.

## HPV negative cancers

The Belgian cancer register looked at all the performed HPV tests before or at the moment of cancer diagnosis (Annual Report, 2017). HPV results after the diagnosis (incidence date) were not included. A tumor was considered HPV positive if at least 1 positive HPV result was registered, regardless of the timespan between the HPV test and the incidence date and irrespectively of previous HPV texts (Annual report, 2017). Only 29% (136 / 468) of invasive cervical cancers (time period 2013- 2014) had some kind of HPV test. Twenty out of 136 (14,7%) of individuals tested had a HPV negative tumor. These figures sound interesting, but they are not conclusive for the entire female population. In modern communication this would probably be called “Fake News”. In Belgium there is only a reimbursement for HPV analysis in case of ASCUS. It is therefore unclear why these women had a HPV analysis. For instance: “Were these women screened as part of a study?” or “Were there other reasons to perform a HPV test in this group?”

From a test point of view, there are multiple questions regarding this representation. For instance, it is unclear which test was used and if all these analyses were done on a smear or on a tissue sample. It is likely that a mix of different HPV tests was used. Also, it remains unknown if these tests were validated. The figure of 14,7% HPV negative cervical cancer mentioned in the Annual Report (period 2013-2014) should be compared with previous data form our country and worldwide (Annual Report, 2017). The first study that reported HPV data in Belgian women with cervical cancer looked at a period before the year 2000. In this study there were 13% HPV “negative” cancers (Tjalma et al., 2001). In an additional study looking at the period 2001-2008 the reported number of HPV negative tumors was 7,1% (Tjalma et al., 2015). Looking at HPV type-specific prevalence data published from 1990 to 2010 (243 studies and 30,848 women with an invasive cervical cancer) a similar decrease in HPV negative tumors was also seen (Li et al., 2011). In this meta-analysis the HPV positivity for the period 1990-1999, 2000-2005 and 2006-2010 was, respectively, 85,9%, 87,9% and 92,9% (Li et al., 2011). The decrease in HPV negative cancers is likely to reflect improvements in the HPV detection methods. The exclusion of misclassified disease by a careful central histopathological review of all samples also reduces the number of HPV negative tumors (Tjalma et al., 2012). Worldwide it is estimated that the percentage for HPV negative tumors fluctuates between 7 – 11% (Clifford et al., 2003; Guan et al., 2012; Hopenhayn et al., 2014; Insinga et al., 2008; Li et al., 2011; Petry et al., 2017).

## Explanations for HPV negative cancers

### Cervical cancers independent of HR HPV

Invasive cervical cancer (ICC) can be divided in three groups. The first group are squamous cervical cancers (SCC) and they account for 75-90% of ICCs (Seoud et al., 2011). The second group are adenocarcinomas (ADC) and adenosquamous cell carcinomas (ASC) which account for 10-25% of all ICCs (Seoud et al., 2011). The ASC has features of both SCC and ADC, but are often included in ADC classification because of their relatively small numbers. The last group is a collection of rare cancers and includes melanoma, sarcoma, lymphoma, neuroendocrine tumors and cancers of unspecified histology (Seoud et al., 2011; Trinh et al., 2004).

There are several reasons for HPV negative cervical cancers (Table I). First of all, there are cervical cancers independent from HPV infection, these are the true HPV negative cancers. For a squamous cancer to be HPV negative is very uncommon (almost 100% HPV positive) (Pirog, 2017). Among adenosquamous cancers the HPV positivity is almost 86% (Holl et al., 2015). In situ adenocarcinomas are almost always high risk HPV positive (Pirog, 2017). The prevalence of HPV among adenocarcinoma varies between the subtypes. ADCs can be divided in a group of subtypes which have a high HPV prevalence and in a group with low HPV prevalence (Table II).

**Table I:** Explanations for HPV negative cancers.

Cervical cancers independent of HR (high risk) HPV	
Cervical cancers who lose the expression of HPV	
Cervical cancer due to low-risk and intermediate risk HPV	
Misclassified cancers	
	Uterine endometrioid adenocarcinoma
	Metastasis from other primary tumors
HPV testing method	

**Table II:** HPV prevalence in adenocarcinoma (Holl et al. 2015; Pirog 2017; Pirog et al. 2018; Sal et al. 2016) ^Inc. on behalf of UICC.Cervical glandular neoplasias (CGN^

ADC		% of ADC	% HPV positive
Usual type		75	80 - 100
Intestinal		8	83 - 100
Villoglandular^*^		3-6	100
Signet ring cell		rare	100
Endometrioid^**^		rare	100
	- From SCJ		100
	- From upper cervix/lower uterine		0
Serous		very rare	30
Clear cell		2-7	28
Gastric type		unknown	0
Mesonephric		rare	0

*villogladular ADC is a well differentiated variant of endocervical, endometrioid, or intestinal ADC. ** Endometrioid ADC is a rare variant developing from either tuboendometrioid metaplasia or endometriosis. The can be divided in tumors originating from the squamous columnar junction zone or from the upper part of the endocervix and lower uterine segment.

Subtypes with a high prevalence of HPV are the usual type, intestinal, villoglandular, signet-ring cell and the endometrioid ADCs which originate from the cervical squamous columnar junction zone. Together they account for more than 90% of all ADCs. Subtypes with a low HPV prevalence are serous and clear cell ADCs. Due to the rareness of these subtypes only a low number of cases are tested. When comparing the different small studies, you notice that the HPV positivity figure vary, which is confusing. Subtypes which are typically HPV negative (unrelated to HPV) are the gastric type, the mesonephric type and the endometrioid ADC from the upper part of the endocervix and lower uterine segment. These subtypes are however rare. The minimal deviation adenocarcinoma of mucinous type is considered a well-differentiated form of gastric ADC (Kojima et al., 2007; Pirog, 2017). The incidence of the gastric type is actually unknown as it is a new histopathologic entity since 2014 (Kojima et al., 2007; Pirog, 2017). The pathogenesis in the group of cervical cancers, with low or no HPV prevalence is unrelated or independent of HPV.

Support for these HPV independent pathway(s) are the fact that these types have been linked to mutations. In clear cell ADC the PI3K-AKT pathway could be involved as in 50% of cases a positive p-AKT and p-mTOR immunostaining is observed (Ueno et al., 2013; Pirog, 2017). In older patients suffering from this subtype of ADC there is a loss of PTEN expression in 50% of cases and an increased expression of EGFR and HER2 in 75% and 50% of the cases, respectively (Ueno et al., 2013; Pirog, 2017). The gastric types are associated to somatic and germ line (Peutz-Jeghers syndrome) STK11 mutations and TP53 mutations (Pirog et al., 2018). In mesonephric ADCs 81% had a KRAS or NRAS mutations, 62% had an ARID1A or ARID1B or SMARCA4 mutations and 0% had a PIK3CA or PTEN mutations (Mirkovic et al., 2015; Pirog, 2017). Mesonephric ADC are characterized by molecular alterations that differ from common variants of cervical adenocarcinoma, which harbor KRAS/NRAS mutations in 7% of cases (Mirkovic et al., 2015). A treatment option for the mesonephric ADC could therefore be inhibitors of the RAS/ MAPK pathway.

### Cervical cancers which lose expression of HPV

Another group of tumors which can be regarded as false negative, are those tumors that lose HPV. More and more reports are describing the molecular features of cervical cancer (Akagi et al., 2014; Ojesina et al., 2014; Banister et al., 2017; Cancer Genome Atlas Research Network et al., 2017). There is a subset of tumors, which no longer express HPV E6/E7 oncogenes (HPV-inactive) (Banister et al., 2017). These HPV-inactive tumors have a global decrease in DNA methylation and an increased WNT/ β-catenin and Sonic Hedgehog signaling (Banister et al., 2017). The somatic mutation landscapes between the HPV active and inactive tumors is significantly different. The HPV- inactive tumors have non-synonymous somatic mutations (specifically targeting TP53, ARID, WNT, and PI3K pathways) and the HPV-active tumors have only few somatic mutations. These altered pathways in HPV inactive cancers open the option for targeted therapies (Banister et al., 2017). More research is needed to explore these treatment options. Treatment strategies focusing on WNT, PI3K, or TP53 mutations may be effective against HPV-inactive tumors and may improve the survival rate in patients suffering from these cervical cancers (Banister et al., 2017).

### Cervical cancer due to non-high risk HPV

To our knowledge, only one publication links cervical cancer and low risk HPV 6 (González- Bosquet et al., 2006). Whether this is the cause or an accidental infection remains unknown. Only a few limited reports link non-high risk HPV types to cervical cancer (Zappacosta et al., 2014; Petry et al., 2017). These estimate that about 1‒2% of primary cervical cancers are associated with non- high risk HPV (Petry et al., 2017). Currently used and validated HPV screening tests are designed to detect high risk HPV only. Therefore, the later fail to detect non-high risk HPV and subsequently their associated cancers.

### Misclassified cancers

Additionally, a group of misclassified endometrial cancers (direct extension) and metastasis from other primary tumors which test HPV negative should be considered. Central pathology reviews in major studies has reclassified multiple cancers (Tjalma et al., 2012; Pirog et al., 2014; Holl et al., 2015). Unfortunately, not all published studies have had a centralized pathology review. In a recent study among HPV negative adenocarcinoma it was not possible in more than 50 % of the cases to determine the origin of the tumor (cervical vs uterine) based upon histological features (Hopenhayn et al., 2014). To differentiate between a primary endometrial adenocarcinoma and a primary adenocarcinoma of the (endo) cervix, based on histological features alone, is difficult if not impossible in some cases. HPV testing together with immunostaining of tumor and stromal cells, can be very useful for this distinction (Pirog, 2017). The combination of ER-; PR-; vimentin -; p16 diffuse +; CEA +; CD 10-; CD 34+ and HPV + is suggestive for an endocervical adenocarcinoma, while the combination of ER+; PR+; vimentin +; p16 patchy +; CEA -; CD 10+; CD 34- and HPV – support the diagnosis of uterine adenocarcinoma. Age is also a helpful indicator. The triad, older age, HPV negative and non-squamous cancer is the signature of an uterine cancer and not of a cervical cancer.

Metastasis in an extra genital site to the cervix is a rare event (Karpathiou et al., 2018). An old study revealed that only 3.7% of the metastatic female genital neoplasms involved the cervix (Mazur et al., 1984).

### HPV testing

There is a difference in HPV prevalence between squamous cancers and ADC. This is due to the fact that some ADC subtypes have low or no HPV positivity. But, undoubtedly it also involves the fact that the HPV DNA load in an ADC is much lower, making its detection a difficult challenge (Pirog, 2017). In contrast to a squamous epithelium, a glandular epithelium does not support a productive HPV infection. Thus, in cases of squamous carcinoma this may lead to a highly replicated episomal HPV DNA copy numbers along with integrated virus in the infected cells. In the glandular epithelium there is no accumulation of replicated episomal HPV DNA in the infected glandular cell and only low copy numbers of HPV DNA are integrated and only a low copy number of HPV DNA present integrated into the cell genome (Pirog et al.,2014). This is due to highly sensitive PCR primers identifying more and different HPV infections (Pirog, 2017).

The first reason for false negative tests are sampling errors. For instance, inadequate cellularity (a cancer with necrosis and/or inflammation is often false negative), obscuring blood or lubricants, inflammation, fixation or cytolysis may result in false negatives. Thus, multiple reasons for questioning the representativeness of HPV testing in currently published studies exist. Some of these studies tested HPV on old (if not very old) stored HPV material, rendering impossible to determine if this material could be representative. A retrospective study showed that tumors from older patients and tumour samples stored for a longer time had a lower HPV prevalence (Pirog et al., 2014). Samples stored for more than 30 years have a significantly lower HPV detection rate (Pirog et al., 2014). The impact is higher in adenocarcinomas than in squamous carcinomas. Other important feature to be considered is the time between excision, fixation and the type of fixing fluid used. In a retrospective study, the use of non-buffered formalin add in different fixation protocols was a significant factor for a decreased detection of HPV (Pirog et al., 2014). In the reported studies, HPV was sometimes tested on archived tissue, fresh-frozen tissue or in liquid biopsy samples. It is assumed that the outcome of these HPV tests, regardless of the type of biopsy, is comparable. However, this is not true. A study revealed that for different adenocarcinomas, HPV positivity in freshly frozen tissue was 14.3% higher than the HPV positivity found in paraffin embedded tissue (the difference was not statistically significant) (Odida et al., 2010). The same study showed that for squamous carcinoma the HPV positivity in paraffin embedded tissue was 1.6% higher than in freshly frozen tissue (the difference was not statistically significant)(Odida et al., 2010). In these studies different HPV tests were used and not all HPV tests were validated. It has to be underlined that all HPV tests are different. For instance, the global difference between HPV 16 and HPV 18 tested by L1 HPV test and E6/E7 HPV test is 91.7% and 72.1%, respectively. This means that 8.3% of HPV 16 and 27.9% of HPV 18 are missed by the L1 HPV test (Tjalma and Depuydt, 2013). Clinicians are generally not aware that there is a huge difference among HPV tests (Tjalma and Depuydt, 2013). In the era of highly effective prophylactic HPV vaccines, it will become important to test for rare HPV types.

It is very likely that false negative results arise due to way tissues were collected, stored and the used HPV test(s). In order to reduce the number of false negative results, it is necessary to standardize an operating procedure (SOP) for tissue collection and HPV testing. The used HPV tests should be validated and have a very high sensitivity. Special attention to the minimal viral load cutoff should be given, because low level persistent HPV infections exist (Weaver et al., 2011). Laboratories performing HPV testing should be accredited by authorized accreditation bodies and in compliance with international standards (Vassilakos et al., 2017).

When a cervical tumor tests negative for HPV, the clinician should have two considerations. First, “could this be a secondary malignancy, either by direct grow or metastasis?”. In some clinical settings almost 68% of negative cervical cancers appeared to be misdiagnosed primary cervical cancers (Petry et al., 2017). Second, “should the sample be re-tested with a different HPV test?”. A HPV-positive result can be explained either by the failure of the initial test procedure to detect high-risk HPV subtypes or by infection with other HPV subtypes, not identified by a standard HPV test (Petry et al., 2017). This approach will reduce the number of mistreatments.

### Screening

The sensitivity in cytology screening ranges between 50 to 70%. This means that 50 to 30% of abnormal lesions are missed. The sensitivity when testing for DNA on high risk HPV in RCTs (Randomized Controlled Trials) is more than 90% (Ronco et al., 2014; Tjalma, 2014), meaning that less than 10% will be missed. The RCTs showed that in the first screening round more high grade lesions were detected with HPV compared to cytology alone. In the following screening rounds no cervical cancer was found in the HPV arm but only in the cytology arm (Gage et al., 2014; Kitchener et al., 2009; Naucler et al., 2007; Rijkaart et al., 2012; Ronco et al., 2010, 2014; Tjalma, 2014; Wright et al., 2015). The end result was a (significant) reduction of cervical cancer in the HPV arm compared to the cytology arm. Worldwide, several countries such as Australia, the UK and the Netherlands, have already switched to primary HPV screening. A large number of countries are considering this transition. Screening with HPV testing is superior to screening by cytology alone. When you compare the different screening methods you will come to the following predictions: no screening will give 8.34 deaths per 1000 women, cytology screening every 3 years will give 0.76 death per 1000 women, primary HR HPV testing or co-testing every 5 years starting at the age of 30 years will give respectively 0.29 and 0.30 cervical cancer deaths per 1000 women (Kim et al., 2018; Learman and Garcia, 2018). All screening methods however will miss cancers. The question is how many?

There is a group of cancers which has a false diagnosis and group of cervical cancers which was missed (false negative) due to the test which was used, not representative sample, no endocervical cells, low viral load, or because of a small amount of DNA. Retrospective analyses without surgical staging overestimate the proportion of HPV negative cervical cancers (Petry et al., 2017). This number should decrease due to the quality control and the improvement of HPV tests.

There is, however, a very small proportion of mainly rare adenocarcinomas which are HPV negative (true negative). A recent study which used next-generation sequencing to characterize primary cervical cancers, found that only 5% of cervical cancers were HPV-negative (Cancer Genome Atlas Research Network et al., 2017). HPV vaccination and primary HPV screening will not prevent or detect these tumors. The cytological accuracy for detecting these lesions remains unknown. These tumors have another pathogenesis. Additional investigations regarding their signaling pathways is needed. This could allow the identification of specific molecular tests, for early detection of these rare tumors. HPV inactive tumors have gene expression, DNA methylation and somatic mutation signatures which are different from HPV- active tumors, and similar to those from other viral- independent cancers (Banister et al., 2017). The latter opens the possibility for specific targeted therapies which may lead to better survival rates. Finally, the incidence of HPV negative tumors does not inform about the performance of primary HPV screening. Data from randomized controlled trials are very clear: If a woman has a normal cytology and no HPV infection or normal cytology and HPV infection, then, her chances of developing a CIN3 + lesion after 5 years is 0,18% and 6,11%, respectively (Arbyn et al., 2012).

Primary HPV screening with dual-stain cytology in Belgium would reduce the incidence of cervical cancer by 36% and the annual cervical mortality by 40% (Tjalma, 2017; Tjalma et al., 2017). We need to improve the screening as we are entering an era of HPV vaccinated women who will get screened. Even though HPV negative cancers could be miss- reported, HPV screening, preferable with triage markers, remain superior to cytology.

Finally, the fact that there are HPV negative cancers should not undermine all idea’s regarding primary HPV screening.

## References

[1] Akagi K, Li J, Broutian TR (2014). Genome-wide analysis of HPV integration in human cancers reveals recurrent, focal genomic instability.. Genome Res.

[2] Annual Report 2017. http://www.bevolkingsonderzoek.be

[3] Arbyn M, Ronco G, Anttila A (2012). Evidence Regarding Human Papillomavirus Testing in Secondary Prevention of Cervical Cancer. Vaccine.

[4] Banister CE, Liu C, Pirisi L (2017). Identification and characterization of HPV-independent cervical cancers. Oncotarget.

[5] (2017). Integrated genomic and molecular characterization of cervical cancer. Nature.

[6] Clifford GM, Smith JS, Plummer M (2003). Human papillomavirus types in invasive cervical cancer worldwide: a meta-analysis. Br J Cancer.

[7] Gage JC, Schiffman M, Katki HA (2014). Reassurance against future risk of precancer and cancer conferred by a negative human papillomavirus test.. J Natl Cancer Inst.

[8] Garland SM, Kjaer SK, Muñoz N (2016). Impact and Effectiveness of the Quadrivalent Human Papillomavirus Vaccine: A Systematic Review of 10 Years of Real-world Experience.. Clin Infect Dis.

[9] González-Bosquet E, Muñoz A, Suñol M (2006). Cervical cancer and low-risk HPV; a case report.. Eur J Gynaecol Oncol.

[10] Guan P, Howell-Jones R, Li N (2012). Human papillomavirus types in 115,789 HPV-positive women: a meta-analysis from cervical infection to cancer.. In J Cancer.

[11] Holl K, Nowakowski AM, Powell N (2015). Human papillomavirus prevalence and type-distribution in cervical glandular neoplasias: Results from a European multinational epidemiological study. Int J Cancer.

[12] Hopenhayn C, Christian A, Christian WJ (2014). Prevalence of human papillomavirus types in invasive cervical cancers from 7 us cancer registries before vaccine introduction. J Low Genit Tract Dis.

[13] Insinga RP, Liaw K-L, Johnson LG (2008). A systematic review of the prevalence and attribution of human papillomavirus types among cervical, vaginal, and vulvar precancers and cancers in the United States.. Cancer Epidemiol Biomarkers Prev.

[14] Karpathiou G, Chauleur C, Hathroubi S (2018). Secondary Tumors of the Gynecologic Tract. Int J Gynecol Pathol.

[15] Kavanagh K, Pollock KG, Cuschieri K (2017). Changes in the prevalence of human papillomavirus following a national bivalent human papillomavirus vaccination programme in Scotland: a 7-year cross-sectional study. Lancet Infect Dis.

[16] Kim JJ, Burger EA, Regan C, Sy S (2018). Screening for Cervical Cancer in Primary Care: A Decision Analysis for the US Preventive Services Task Force. JAMA.

[17] Kitchener HC, Almonte M, Thomson C (2009). HPV testing in combination with liquid-based cytology in primary cervical screening (ARTISTIC): a randomised controlled trial.. Lancet Oncol.

[18] Kojima A, Mikami Y, Sudo T (2007). Gastric morphology and immunophenotype predict poor outcome in mucinous adenocarcinoma of the uterine cervix.. Am J Surg Pathol.

[19] Learman LA, Garcia FAR (2018). Screening for Cervical Cancer: New Tools and New Opportunities. JAMA.

[20] Li N, Franceschi S, Howell-Jones R (2011). Human papillomavirus type distribution in 30,848 invasive cervical cancers worldwide: Variation by geographical region, histological type and year of publication. Int J Cancer.

[21] Mazur MT, Hsueh S, Gersell DJ (1984). Metastases to the female genital tract. Analysis of 325 cases.. Cancer.

[22] Mirkovic J, Sholl LM, Garcia E (2015). Targeted genomic profiling reveals recurrent KRAS mutations and gain of chromosome 1q in mesonephric carcinomas of the female genital tract.. Mod Pathol.

[23] Naucler P, Ryd W, Törnberg S (2007). Human papillomavirus and Papanicolaou tests to screen for cervical cancer.. N Engl J Med.

[24] Odida M, de Sanjose S, Sandin S (2010). Comparison of human papillomavirus detection between freshly frozen tissue and paraffin embedded tissue of invasive cervical cancer. Infect Agent Cancer.

[25] Ojesina AI, Lichtenstein L, Freeman SS (2014). Landscape of genomic alterations in cervical carcinomas. Nature.

[26] Petry KU, Liebrich C, Luyten A (2017). Surgical staging identified false HPV-negative cases in a large series of invasive cervical cancers.. Papillomavirus Res.

[27] Pirog EC (2017). Cervical Adenocarcinoma: Diagnosis of Human Papillomavirus–Positive and Human Papillomavirus– Negative Tumors.. Arch Pathol Lab Med.

[28] Pirog EC, Lloveras B, Molijn A (2014). HPV prevalence and genotypes in different histological subtypes of cervical adenocarcinoma, a worldwide analysis of 760 cases.. Mod Pathol.

[29] Pirog EC, Park KJ, Kiyokawa T (2018). Gastric-type Adenocarcinoma of the Cervix: Tumor With Wide Range of Histologic Appearances.. Adv Anat Pathol.

[30] Rijkaart DC, Berkhof J, Rozendaal L (2012). Human papillomavirus testing for the detection of high-grade cervical intraepithelial neoplasia and cancer: final results of the POBASCAM randomised controlled trial.. Lancet Oncol.

[31] Ronco G, Dillner J, Elfström KM (2014). Efficacy of HPV- based screening for prevention of invasive cervical cancer: follow-up of four European randomised controlled trials. Lancet.

[32] Ronco G, Giorgi-Rossi P, Carozzi F (2010). Efficacy of human papillomavirus testing for the detection of invasive cervical cancers and cervical intraepithelial neoplasia: a randomised controlled trial.. Lancet Oncol.

[33] Sal V, Kahramanoglu I, Turan H (2016). Primary signet ring cell carcinoma of the cervix: A case report and review of the literature.. Int J Surg Case Rep.

[34] Seoud M, Tjalma WA, Ronsse V (2011). Cervical adenocarcinoma: Moving towards better prevention. Vaccine.

[35] Suárez-Peñaranda JM, Abdulkader I, Barón-Duarte FJ (2007). Signet-ring cell carcinoma presenting in the uterine cervix: report of a primary and 2 metastatic cases.. Int J Gynecol Pathol.

[36] Tjalma WA, Weyler JJ, Bogers JJ (2001). The importance of biological factors (bcl-2, bax, p53, PCNA, MI, HPV and angiogenesis) in invasive cervical cancer.. Eur J Obstet Gynecol Reprod Biol.

[37] Tjalma WA, Arbyn M, Paavonen J (2004). Prophylactic human papillomavirus vaccines: The beginning of the end of cervical cancer. Int J Gynecol Cancer.

[38] Tjalma WA, Van Waes TR, Van den Eeden LEM (2005). Role of human papillomavirus in the carcinogenesis of squamos cell carcinoma and adenocarcinoma of the cervix.. Best Pract Res Clin Obstet Gynaecol.

[39] Tjalma WA, Depuydt CE (2013). Eur J Obstet Gynecol Reprod Biol. Cervical cancer screening: Which HPV test should be used - L1 or E6/E7.

[40] Tjalma WA (2014). The ideal cervical cancer screening recommendation for Belgium, an industrialized country in Europe.. Eur J Gynaecol Oncol.

[41] Tjalma WA (2015). There are two prophylactic human papillomavirus vaccines against cancer, and they are different.. J Clin Oncol.

[42] Tjalma WA, Trinh XB, Rosenlund M (2015). A cross-sectional, multicentre, epidemiological study on human papillomavirus (HPV) type distribution in adult women diagnosed with invasive cervical cancer in Belgium.. Facts Views Vis ObGyn.

[43] Tjalma WA (2017). Diagnostic performance of dual-staining cytology for cervical cancer screening: A systematic literature review.. Eur J Obstet Gynecol Reprod Biol.

[44] Tjalma WA, Kim E, Vandeweyer K (2017). The impact on women’s health and the cervical cancer screening budget of primary HPV screening with dual-stain cytology triage in Belgium.. Eur J Obstet Gynecol Reprod Biol.

[45] Tjalma WA, Brasseur C, Top G (2018). HPV vaccination coverage in the federal state of Belgium according to regions and their impact.. Facts Views Vis ObGyn.

[46] Trinh XB, Bogers JJ, Van Marck EA (2004). Treatment policy of neuroendocrine small cell cancer of the cervix.. Eur J Gynaecol Oncol.

[47] Ueno S, Sudo T, Oka N, Wakahashi S (2013). Absence of human papillomavirus infection and activation of PI3K-AKT pathway in cervical clear cell carcinoma. Int J Gynecol Cancer.

[48] Vassilakos P, Tran PL, Sahli R (2017). Swiss Med Wkly. HPV-negative CIN3 and cervical cancer in Switzerland: any evidence of impact on screening policies.

[49] Weaver B, Shew M, Qadadri B (2011). Low-level persistence of human papillomavirus 16 DNA in a cohort of closely followed adolescent women.. J Med Virol.

[50] Wright TC, Stoler MH, Behrens CM (2015). Primary cervical cancer screening with human papillomavirus: end of study results from the ATHENA study using HPV as the first-line screening test.. Gynecol Oncol.

[51] Zappacosta R, Lattanzio G, Viola P (2014). A very rare case of HPV-53-related cervical cancer, in a 79-year-old woman with a previous history of negative Pap cytology. Clin Interv Aging.

